# A review on delayed presentation of diaphragmatic rupture

**DOI:** 10.1186/1749-7922-4-32

**Published:** 2009-08-21

**Authors:** Farhan Rashid, Mallicka M Chakrabarty, Rajeev Singh, Syed Y Iftikhar

**Affiliations:** 1 Division of GI Surgery, University of Nottingham, Graduate Entry Medical School, Uttoxeter Road, Derby, DE22 3DT, UK; 2 Department of Upper GI Surgery, Royal Derby Hospital, Uttoxeter Road, Derby, DE22 3NE, UK; 3 Department of Radiology, Royal Derby Hospital, Uttoxeter Road, Derby, DE22 3NE, UK

## Abstract

Diaphragmatic rupture is a life-threatening condition. Diaphragmatic injuries are quite uncommon and often result from either blunt or penetrating trauma. Diaphragmatic ruptures are usually associated with abdominal trauma however, it can occur in isolation. Acute traumatic rupture of the diaphragm may go unnoticed and there is often a delay between the injury and the diagnosis. A comprehensive literature search was performed using the terms "delayed presentation of post traumatic diaphragmatic rupture" and "delayed diaphragmatic rupture". The diagnostic and management challenges encountered are discussed, together with strategies for dealing with them. We have focussed on mechanism of injury, duration, presentation and site of injury, visceral herniation, investigations and different approaches for repair. We intend to stress on the importance of delay in presentation of diaphragmatic rupture and to provide a review on the available investigations and treatment methods. The enclosed case report also emphasizes on the delayed presentation, diagnostic challenges and the advantages of laparoscopic repair of delayed diaphragmatic rupture.

## Review of Literature

A Pubmed search was conducted using the terms "delayed presentation of post traumatic diaphragmatic rupture" and "delayed diaphragmatic rupture". Although quite a few articles were cited, the details of presentation, investigations and treatment discussed in each of these were not identical, accounting for the variation in the data presented below.

Late presentation of diaphragmatic rupture is often a result of herniation of abdominal contents into the thorax[1]. Sudden increase in the intra abdominal pressure may cause a diaphragmatic tear and visceral herniation[2]. The incidence of diaphragmatic ruptures after thoraco-abdominal traumas is 0.8–5% [3] and up to 30% diaphragmatic hernias present late[4]. Diaphragmatic, lumbar and extra-thoracic hernias are well described complications of blunt trauma [5]. Incorrect interpretation of the x ray or only intermittent hernial symptoms are frequent reasons for incorrect diagnosis[6].

## Mechanism of injury

Diaphragmatic rupture with abdominal organ herniation was first described by Sennertus in 1541[7,8]. Diaphragmatic injury is a recognised consequence of high velocity blunt and penetrating trauma to the abdomen and chest rather than from a trivial fall[8]. These patients usually have multi system injuries because of the large force required to rupture the diaphragm[9].

Blunt trauma to the abdomen increases the transdiaphragmatic pressure gradient between the abdominal compartment and the thorax[10]. This causes shearing of a stretched membrane and avulsion of the diaphragm from its points of attachments due to sudden increase in intra abdominal pressure, transmitted through the viscera[11]. Delay in presentation of a diaphragmatic hernia could be explained by various different hypotheses. Delayed rupture of a devitalised diaphragmatic muscle may occur several days after the initial injury [8]. This is best exemplified in the case report of bilateral diaphragmatic rupture [12], where the left diaphragmatic rupture was identified 24 hours after the motor vehicle accident and the right diaphragm, which was explored at the initial laparotomy, manifested 10 days later. Intra operative findings at the right thoracotomy revealed thin, inflamed diaphragm with necrotic muscle. The devitalised diaphragmatic muscle continues as a barrier until the inflammatory process weakens it [12]. Extubation precipitates this phenomenon when the intrathoracic pressure becomes negative[9]. However the more likely explanation is a possible delayed detection assuming that the diaphragmatic defect occurring with injury manifests only when herniation occurs[9]. Traumatic diaphragmatic hernia is a frequently missed diagnosis and there is commonly a delay between trauma and diagnosis[13].

## Duration before presentation

Grimes in 1974[14] described the 3 phases of the rupture of the diaphragm. The acute phase is at the time of the injury to the diaphragm. The delayed phase is associated with transient herniation of the viscera thus accounting for absence or intermittent non specific symptoms. The obstruction phase signifies complication of a long standing herniation, manifesting as obstruction, strangulation and rupture[8]. The systematic review of the literature suggests 1 case being reported at 24 hours following trauma[12], 1 case each on Day 9[15], Day10[12] and Day11[8] following trauma. Two cases have been reported 6 months following the trauma [16,17] while 1 case each had been reported 12 months[11], 18 months [3] and 24 months [18] following trauma. Two cases have been reported at 5 years[19,20], 1 case each at 8 years[21], 10 years[7], 20 years[1], 28 years[22], 40 years [13] and 50 years[23].

## Presenting symptom

Due to co existing injuries and the silent nature of diaphragmatic ruptures, the diagnosis can sometimes be missed in the acute phase and may present later on with obstructive symptoms due to incarcerated organs in the diaphragmatic defect [24] or eventual strangulation[7]. Patients present with non specific symptoms and may complain of chest pain, abdominal pain, dyspnoea, tachypnoea and cough [1]. A high index of suspicion, together with the knowledge of the mechanism of trauma, is the key factor for the correct diagnosis[25]. Our literature review confirmed 8 cases presenting acutely with haemodynamic instability with abdominal pain [15,24]. 3 cases were reported to be asymptomatic diaphragmatic hernias [24]. Respiratory distress was the presenting feature in 10 cases [7,11-13,17,21,24]. Abdominal pain was the presenting feature in 3 cases [13,17,18]. The patho-physiology was intestinal obstruction in 11 cases [8,21,24], 1 case of pneumopericarditis [26], 3 cases of tension faeco-pneumothorax [16,19,21]. There is report of one case presenting with hematemeisis and malena [22].

## Site of rupture

Although autopsy studies have revealed equal incidence of right and left diaphragmatic ruptures, antemortum study reports suggest 88–95% of diaphragmatic ruptures occurred on the left side [8]. Right sided ruptures are associated with high mortality and morbidity [16] and thus the under diagnosis of right sided injuries may be due to greater pre hospital mortality [8]. Right sided tears are significantly less likely than left sided tears because of the protective effect of the liver [2,16,27]. This could also be explained by better visualisation of the left diaphragm, on diagnostic laparoscopy, but restricted visualisation of the right diaphragm [28]. The systematic review of literature has confirmed 27 cases of left sided rupture [4,8,11,13,16-19,21,22,24,26,29,30] and 13 cases of right sided rupture were reported [2-4,7,15,24,31-33]. The rarely reported sites include 1 central diaphragmatic hernia [20], 2 bilateral [12,24] and 1 trans-diaphragmatic intercostal hernia [34]

The systematic review of literature also confirmed intra abdominal and retroperitoneal contents in the hernial sac, which are summarised in the table below (Table 1) [35-37].

**Table 1:** Type of visceral herniation

**Sac Contents**	**No of cases**	**References**
Strangulated Transverse Colon	1	[13]

Perforated Transverse Colon	3	[16,19,21]

Splenic flexure	2	[12,18]

Splenic flexure cancer	1	[4]

Intrathoracic Splenosis	2	[8,35]

Spleen	2	[12,22]

Right hepatic lobe	6	[2,7,15,31-33]

Small Bowel	1	[31]

Stomach/Perforated gastric ulcer	6	[8,12,17,26,29,30]

Intra-thoracic gastric volvulus	2	[36,37]

Kidney, Ureter and Renal Vein	1	[7]

Part of Ascending and Transverse Colon	1	[7]

Gall Bladder	1	[7]

Omentum/Mesentery	2	[20,34]

## Investigations

The studies published before 1996 have quoted that 12–69% of diaphragmatic ruptures are missed at the pre operative phase [38-40]. CT scan was not widely used investigation when these papers were published. However, with the introduction of reformatting of images the sensitivity of the CT scan in picking up the diaphragmatic rupture has significantly increased[41]. While audible bowel sounds on the chest auscultation suggests displaced bowel loops, a chest x ray is the first line of investigation, repeated imaging increases sensitivity[8]. Insertion of a naso-gastric tube can decompress the intra-thoracic stomach to delineate a chest x ray interpretation [8,29] and increase the diagnostic sensitivity to approximately 75%[8]. The sensitivity of chest radiographs is 46% for left sided ruptures and 17% for right sided ruptures [42]. Helical CT with axial, sagittal and coronal reconstruction increases the sensitivity to 73% and the specificity to 90%[12]. A diagnostic laparoscopy and/or diagnostic thoracoscopy could be performed as a semi-elective procedure, the timing planned in accordance with the heamodynamic and respiratory status of the patient [27,28]. Meticulous inspection and palpation of the diaphragm should be performed during laparotomy in patients with trauma [12].

The systematic review of literature confirmed chest x ray findings of bowel loops in the left hemithorax [12,13], abundant hydropneumothorax [21], elevation of the left diaphragmatic dome[7,18,33], loculated left pneumothorax [8], mediastinal shift [16], free gas under the diaphragm [18] and subdiaphragmatic densities [18]. The abdominal x ray findings reported features of large bowel obstruction [18]. Contrast X ray has been reported as showing large part of the stomach lying in left chest [17]. Intrapleural herniation of large intestine has been reported as CT scan findings of intrapleural herniation of large intestine and abundant pleural effusion [21], Intrathoracic displacement of liver[12,15,33], intrathoracic spleen with splenic vein thrombosis [22], large right diaphragmatic rupture with herniation of liver, gall bladder, right kidney, ureter and renal vein. Along with distal ascending colon and proximal transverse colon[7], Collar Sign (Waist like constriction) is produced by compression of herniated organs [10,16]. Diaphragmatic discontinuity and dependent viscera sign (abdominal organs set against the posterior ribs) [10,43] have also been reported. Pleuro-pulmonary sonography has been used in one case to confirm condensed lung with pleural effusion along with interruption of right hemidiaphragm with intrathoracic hepatic parenchyma, dilatation of hepatic veins and collapse of IVC with inspiration[15]. Intraperitoneal injection of technetium sulphur colloid can be used to diagnose rupture of right diaphragm[44]. MR scan has been performed and reported displacement of the liver [32].

## Repair of diaphragmatic rupture

Surgical treatment of long-standing post traumatic diaphragmatic rupture is the same as that applicable in diaphragmatic hernias [6]. The first successful repair was performed by Riolfi in 1886[8]. The surgical treatment usually performed includes hernia reduction, pleural drainage and repair of the diaphragmatic defect. This may be performed either through an open laparotomy or thoracotomy or through laparoscopy or thoracoscopy. The mortality from elective repair is low but the mortality from ischaemic bowel secondary to strangulation may be as high as 80%[7] (Table 2) [45].

**Table 2:** Repair of Diaphragmatic rupture

**Surgical Repair**	**No of Cases**	**References**
Laparotomy/Thoraco- laparotomy + Repair	27	[8,12,16,18,20,21,24]

Laparotomy/Thoraco Laparotomy + Repair with synthetic mesh	3	[12,24]

Laparoscopy/Thoracoscopy+Repair	2	[3,17]

Thoracoscopy	1	[15]

Laparoscopy + Repair with synthetic mesh	1	[45]

The Laparoscopic surgery is now widely accepted as a preferable intervention in acute appendicitis, acute cholecystitis and most gynaecological emergencies. Likewise its role in evaluation of diaphragmatic injuries and its repair has been also been suggested. However, this should be carried out with caution and in the presence of required advanced laparoscopic skills[28]. Neugebauer et al, 2006, have also mentioned these advanced laparoscopic procedures have only achieved grade B or C recommendation as compared to laparoscopic interventions for acute cholecystitis or appendicitis which are highly recommended (Grade A, highest grade recommendation) [46]. Thoracoscopic repair of missed diaphragmatic injury has been reported [47]. In addition, thoracolaparoscopic repair of traumatic diaphragmatic rupture has also been recommended provided there is no associated abdominal organ injury [48] However, thoracoscopy sometimes allows repair of only small lesions [49].

Certain problems associated with laparoscopic repair have also been reported [50]. However as described before in the literature[51] and also in the enclosed case report, the laparoscopic repair can be carried out without intraoperative hypoxemia, tension pneumothorax or increased peak airway pressures.

The advantages of using the mesh have been widely discussed in the literature and mesh repair has also been preferred because of the decreased risk of recurrence of the hernias [52,53] In addition, less adhesions have been reported when mesh is placed laparoscopically as compared to their use during open surgery[54].

Laparoscopic repair of diaphragmatic rupture has been carried out in the past [51]. It is difficult to draw conclusion concerning the best approach. However, for procedures like laparoscopic repair of diaphragmatic rupture there is a need for more and better performed controlled clinical trials.

## Our recent experience of delayed diaphragmatic rupture

**Figure 1:**
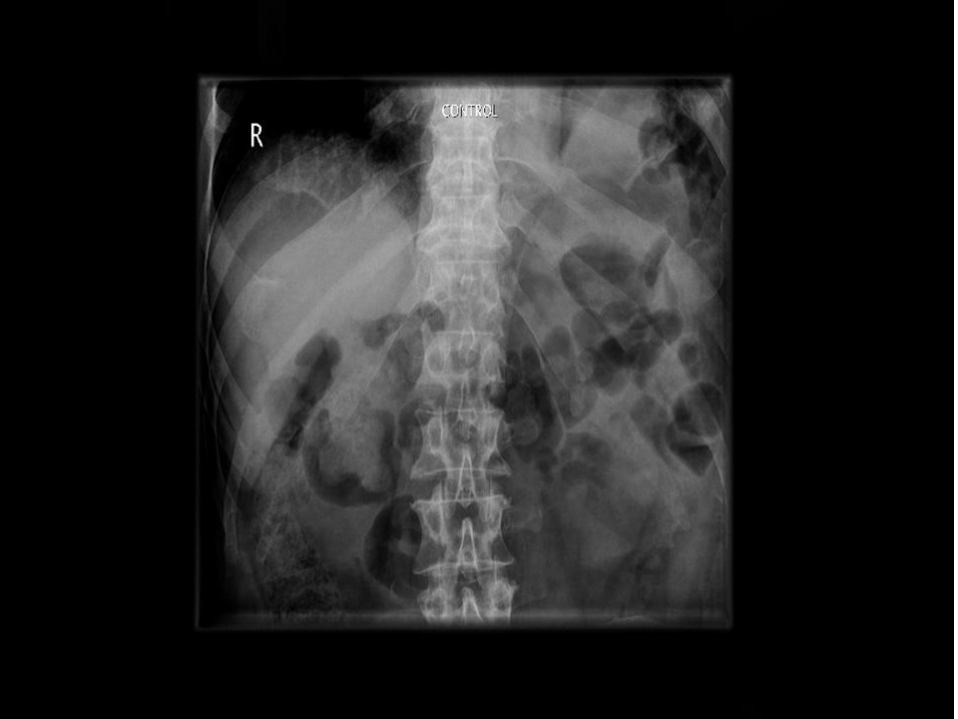
**Plain abdominal x-ray on presentation**. Note nonvisualization of the left hemidiaphragm and bowel gas at the left lung base.

**Figure 2:**
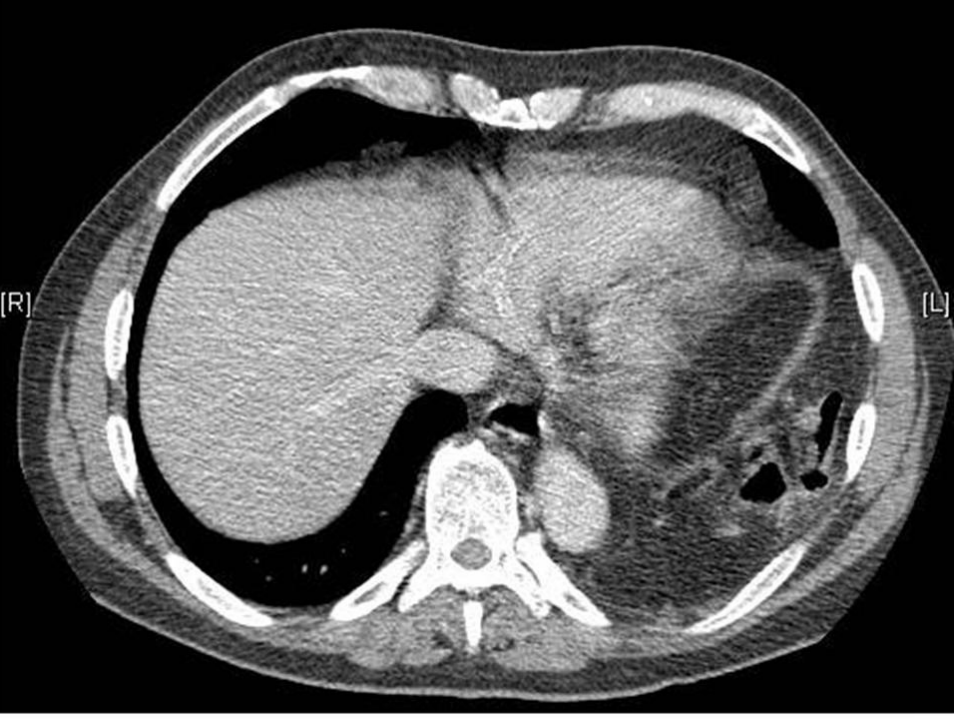
**Axial post IV contrast CT through the lower chest/upper abdomen showing loops of bowel herniating through the disrupted left hemidiaphragm**.

**Figure 3:**
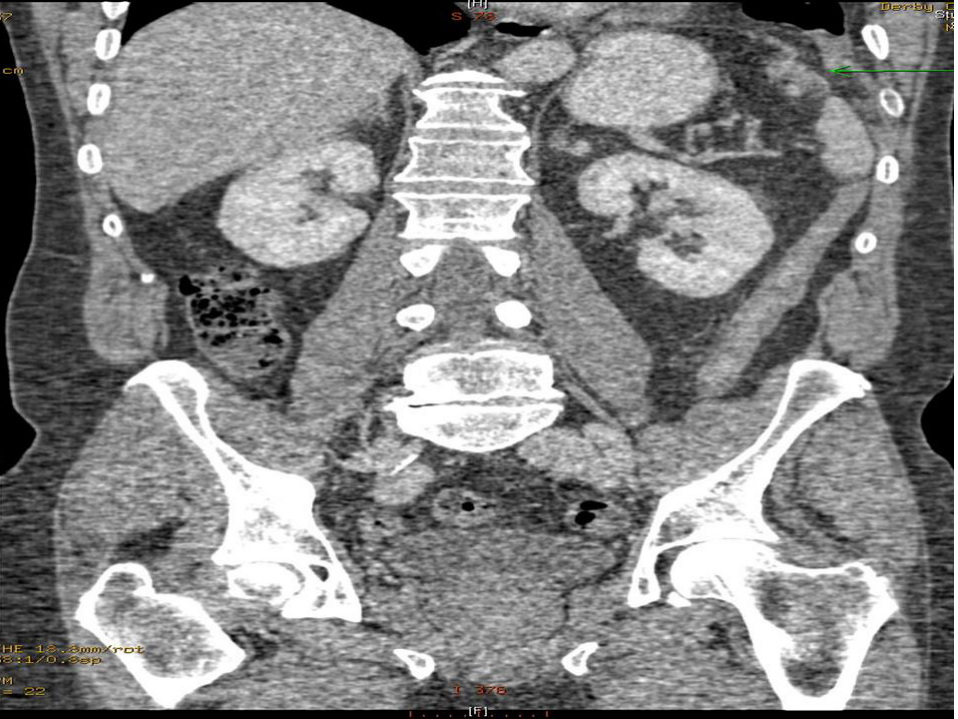
**Coronal CT scan showing disrupted left hemidiaphragm**.

**Figure 4:**
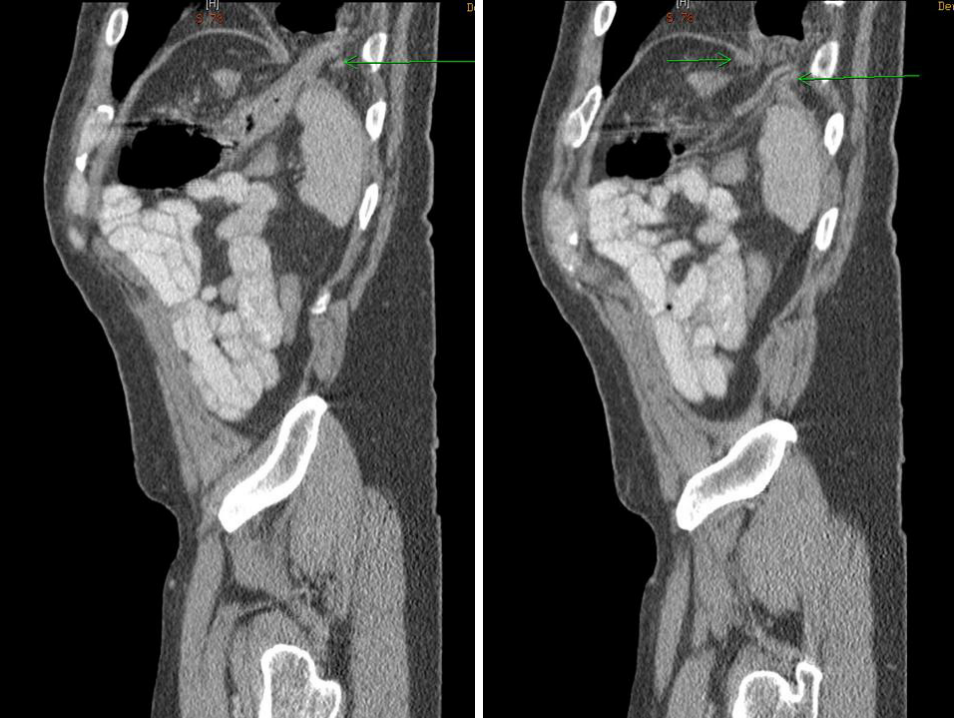
**Saggittal CT showing disrupted left hemidiaphragm with herniation of bowel**.

**Figure 5:**
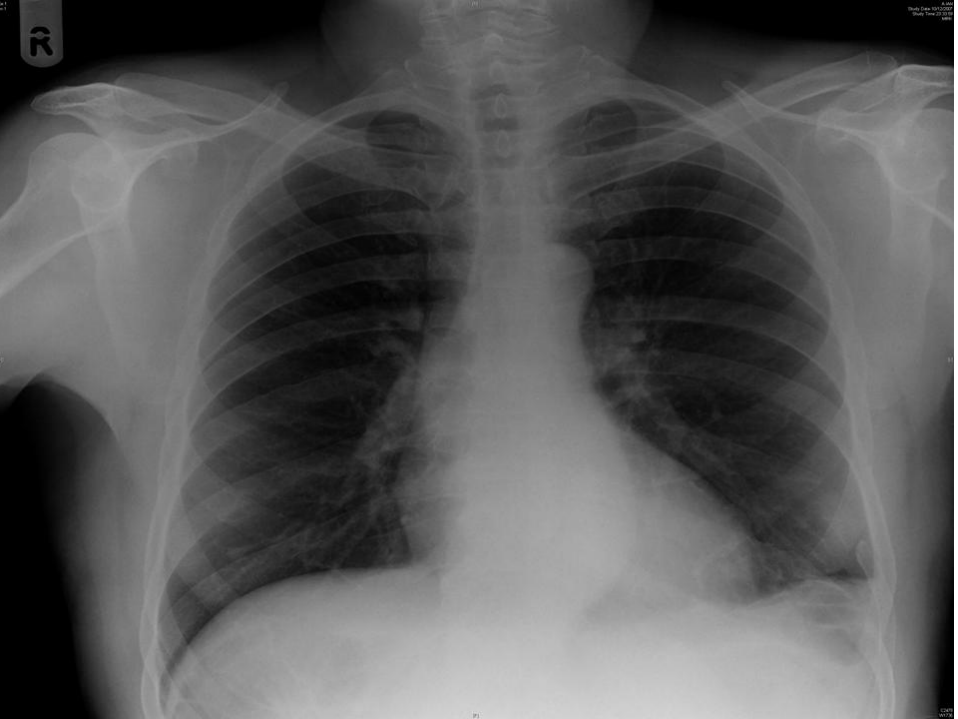
**Previous Chest radiograph with a discontinuous left lower posterior 9th rib**. Note the normal left hemidiaphragm.

A 63 year old man presented with a short history of left sided abdominal associated with nausea. It was colicky in nature and sudden in onset. There was no change in bowel habits. The patient weighed 74 kilograms, with a BMI of 25.6. On examination he was tender in left upper quadrant. He was haemodynamially stable. Baseline blood investigations were inconclusive. X-ray suggested non-visualization of left hemidiaphragm and bowel loops at the left lung base. (Figure 1) The following day he developed persistent pain and vomiting. A CT scan (Figure 2, 3 and 4) were performed and it showed diaphragmatic hernia with colon in left chest. He had a past history of fall at work 9 years ago and had then presented with left flank pain and chest pain on inspiration for 3 days. At that time chest x-ray showed fracture of left lower ribs, along with left sided pleural effusion, which was treated successfully with chest drainage. He also had ultrasound at that time which showed no evidence of splenic injury. In last 9 years he had multiple admissions with similar symptoms and was investigated for renal stones as well. The only available previous chest x-ray showed a normal left hemidiaphragm and discontinuity of the posterior part of the ninth rib. (Figure 5)

Therefore, after confirming the diagnosis of delayed diaphragmatic rupture, the repair of the offending hernia was undertaken laparoscopically. A five port approach was used, employing two 10 mm ports (primary port in the supraumblical position, the other in left midclavicular line two fingers breadth below the costal margin, a 6 mm port in the right mid claviular line two fingers below the costal margin, another port in the left flank and a Nathanson's liver retractor was placed in the epigastric area immediately under the xiphoid process.

**Figure 6:**
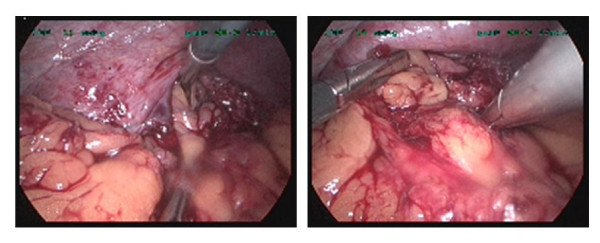
**Intraoperative pictures**.

The key operative findings included omentum and splenic flexure of the colon in the left chest through a previously ruptured diaphragm just lateral and above to the spleen. The lower lobe of the left lung was found to be collapsed. Omentum was dissected off its adhesions and retrieved. The splenic flexure was badly stuck posteriorly, however, was successfully dissected and retrieved into peritoneal cavity. (Figure 6) The repair was performed with interrupted Gortex^® ^sutures. Repair of the remaining defect required porcine mesh of 7 × 10 cm diameter (Surgisis Biodesign, Cook Ireland, Ltd., Limerick, Ireland). These were put in place and secured with protac stapler. A chest drain was also inserted in the left thoracic cavity. The patient remained stable during the intraoperative phase.

**Figure 7:**
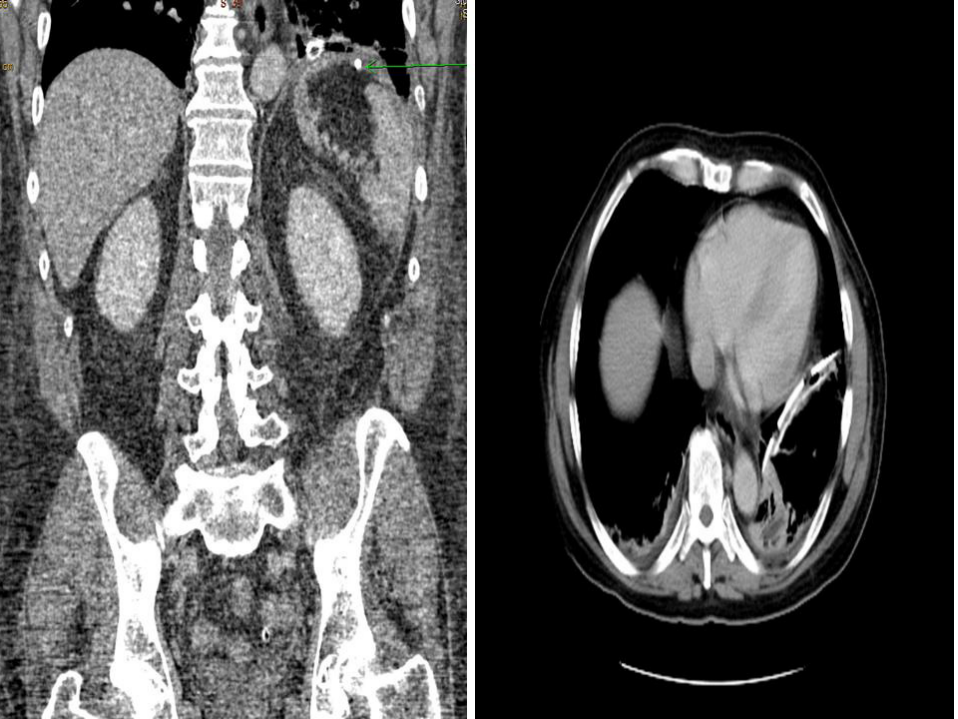
**(a and b): Post operative CT (Coronal and axial views)**. Note the repaired left diaphragam and tip of the chest drain in situ with some patchy basal consolidation (Arrow pointing to protec stapler).

Postoperatively the patient developed minimal left basal consolidation but thereafter he had an uneventful recovery (Figure 7). Later on, he was discharged from the hospital, six days after his operation and was asymptomatic at 6 months follow up.

## Summary

A high clinical index of suspicion is needed to diagnose and effectively manage diaphragmatic rupture even with a remote history of high-velocity injury [55]. This is particularly true when other signs of severe trauma are present such as multiple rib fracture, lacerations of liver and spleen or a history of deceleration injury [2]. Ramdass et all [19] have emphasised that when tension pneumothorax and diaphragmatic hernia coexist, the contents of the visceral sac may be completely reduced and the hernia is thus masked. The drainage of a considerable amount of serous fluid in addition to air, in the presence of tension pneumothorax, may suggest a communication with the peritoneal cavity [19].

We do recommend that a high index of suspicion should be kept in mind while dealing with patients who do get readmitted with upper abdominal symptoms whenever there is a history of trauma or blunt injury regardless of the fact whether it was few days ago or many years ago. We consider laparoscopic repair to be a suitable and safe procedure for treatment of diaphragmatic rupture.

## Competing interests

The authors declare that they have no competing interests.

## Authors' contributions

FR and MMC performed the literature search, extracted the data and wrote the manuscript. RS helped with radiological images. SY Iftikhar performed the operation. FR, MMC, RS and SYI all helped in writing different subsections of the review. All authors contributed to the manuscript, and all read and approved the final version.
